# Single-Cell RNA-Seq Reveals *LRRC75A*-Expressing Cell Population Involved in VEGF Secretion of Multipotent Mesenchymal Stromal/Stem Cells Under Ischemia

**DOI:** 10.1093/stcltm/szad029

**Published:** 2023-06-02

**Authors:** Takumi Miura, Tsukasa Kouno, Megumi Takano, Takuya Kuroda, Yumiko Yamamoto, Shinji Kusakawa, Masaki Suimye Morioka, Tohru Sugawara, Takamasa Hirai, Satoshi Yasuda, Rumi Sawada, Satoko Matsuyama, Hideya Kawaji, Takeya Kasukawa, Masayoshi Itoh, Akifumi Matsuyama, Jay W Shin, Akihiro Umezawa, Jun Kawai, Yoji Sato

**Affiliations:** Division of Cell-Based Therapeutic Products, National Institute of Health Sciences, Kanagawa, Japan; Center for Regenerative Medicine, National Center for Child Health and Development, Tokyo, Japan; RIKEN Center for Integrative Medical Sciences, Yokohama, Japan; Division of Cell-Based Therapeutic Products, National Institute of Health Sciences, Kanagawa, Japan; Division of Cell-Based Therapeutic Products, National Institute of Health Sciences, Kanagawa, Japan; RIKEN Center for Integrative Medical Sciences, Yokohama, Japan; Division of Cell-Based Therapeutic Products, National Institute of Health Sciences, Kanagawa, Japan; RIKEN Center for Integrative Medical Sciences, Yokohama, Japan; Center for Regenerative Medicine, National Center for Child Health and Development, Tokyo, Japan; Biopharmaceutical and Regenerative Sciences, Graduate School of Medical Life Science, Yokohama City University, Yokohama, Japan; Division of Cell-Based Therapeutic Products, National Institute of Health Sciences, Kanagawa, Japan; Division of Cell-Based Therapeutic Products, National Institute of Health Sciences, Kanagawa, Japan; Division of Cell-Based Therapeutic Products, National Institute of Health Sciences, Kanagawa, Japan; Division of Cell-Based Therapeutic Products, National Institute of Health Sciences, Kanagawa, Japan; Center for Reverse TR, Osaka Habikino Medical Center, Osaka Prefectural Hospital Organization, Osaka, Japan; RIKEN Center for Integrative Medical Sciences, Yokohama, Japan; Research Center for Genome & Medical Sciences, Tokyo Metropolitan Institute of Medical Science, Tokyo, Japan; RIKEN Center for Integrative Medical Sciences, Yokohama, Japan; RIKEN Center for Integrative Medical Sciences, Yokohama, Japan; Center for Reverse TR, Osaka Habikino Medical Center, Osaka Prefectural Hospital Organization, Osaka, Japan; RIKEN Center for Integrative Medical Sciences, Yokohama, Japan; Genomic Institute of Singapore, Agency for Science, Technology and Research, Singapore; Center for Regenerative Medicine, National Center for Child Health and Development, Tokyo, Japan; RIKEN Center for Integrative Medical Sciences, Yokohama, Japan; Life Science Technology Project, Kanagawa Institute of Industrial Science and Technology, Kawasaki, Japan; Division of Cell-Based Therapeutic Products, National Institute of Health Sciences, Kanagawa, Japan; Life Science Technology Project, Kanagawa Institute of Industrial Science and Technology, Kawasaki, Japan; Department of Cellular and Gene Therapy Products, Graduate School of Pharmaceutical Sciences, Osaka University, Osaka, Japan

**Keywords:** multipotent mesenchymal stromal/stem cell, single-cell transcriptome analysis, VEGF, LRRC75A, cell subpopulation, biomarker, angiogenic potential

## Abstract

Human multipotent mesenchymal stromal/stem cells (MSCs) have been utilized in cell therapy for various diseases and their clinical applications are expected to increase in the future. However, the variation in MSC-based product quality due to the MSC heterogeneity has resulted in significant constraints in the clinical utility of MSCs. Therefore, we hypothesized that it might be important to identify and ensure/enrich suitable cell subpopulations for therapies using MSC-based products. In this study, we aimed to identify functional cell subpopulations to predict the efficacy of angiogenic therapy using bone marrow-derived MSCs (BM-MSCs). To assess its angiogenic potency, we observed various levels of vascular endothelial growth factor (VEGF) secretion among 11 donor-derived BM-MSC lines under in vitro ischemic culture conditions. Next, by clarifying the heterogeneity of BM-MSCs using single-cell RNA-sequencing analysis, we identified a functional cell subpopulation that contributed to the overall VEGF production in BM-MSC lines under ischemic conditions. We also found that leucine-rich repeat-containing 75A (*LRRC75A*) was more highly expressed in this cell subpopulation than in the others. Importantly, knockdown of *LRRC75A* using small interfering RNA resulted in significant inhibition of VEGF secretion in ischemic BM-MSCs, indicating that *LRRC75A* regulates VEGF secretion under ischemic conditions. Therefore, *LRRC75A* may be a useful biomarker to identify cell subpopulations that contribute to the angiogenic effects of BM-MSCs. Our work provides evidence that a strategy based on single-cell transcriptome profiles is effective for identifying functional cell subpopulations in heterogeneous MSC-based products.

## Graphical Abstract

**Figure:**
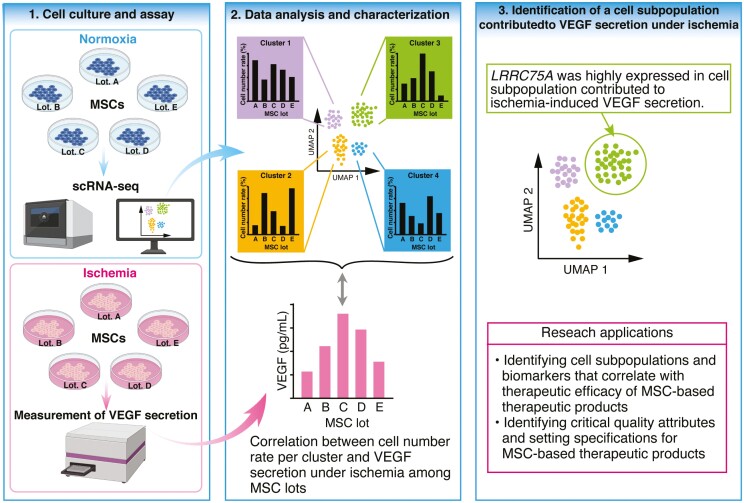
We identified a cell subpopulation associated with VEGF secretion in human multipotent mesenchymal stromal/stem cells under ischemia using single-cell transcriptome analysis and highlighted the potential contribution of LRRC75A on inducing VEGF secretion under ischemia.

**Significance Statement**

We aimed to identify a cell subpopulation useful for the prediction of the efficacy of angiogenic therapy using human MSCs. We assessed VEGF secretion, a potential mechanism of the angiogenic effect, in bone marrow-derived MSCs under ischemia. Significant variations were observed in VEGF production. Single-cell RNA-sequencing analysis identified a functional cell subpopulation whose size was positively correlated with VEGF secretion under ischemia. Furthermore, *LRRC75A* was highly expressed in this subpopulation. Therefore, *LRRC75A* may be a useful biomarker of cell subpopulations with angiogenic potential. Identification of functional cell subpopulations in MSCs will help improve the quality control in manufacturing MSC-based therapeutic products.

## Introduction

Multipotent mesenchymal stromal/stem cells (MSCs) can be isolated from various tissues, including the bone marrow, adipose tissue, umbilical cord, and placenta.^1,2^ Notably, the first MSCs were isolated from the non-hematopoietic component of bone marrow in the 1960s.^3^ The biological functions of MSCs have been characterized, and MSCs have been widely applied in regenerative cell therapy to treat several diseases.^4,5^ The MSC phenotype is determined by their microenvironment. For instance, MSCs obtained from various tissue sources possess different biological characteristics, known as ­tissue-source-related heterogeneity.^6,7^ This heterogeneity causes differences in the biological and functional features among MSC lines, including different growth and proliferative ability and angiogenic, differentiation, and immunomodulatory potential.^8-11^ Moreover, the heterogeneity of MSC-based products makes it difficult to assess comparability among them, which leads to serious consequences in clinical practice.^12,13^ Therefore, to achieve a consistent therapeutic effect by MSC-based products, it is imperative to characterize their heterogeneity and identify cell subpopulations exhibiting clinically relevant features. However, owing to the high cellular heterogeneity of MSCs, it is difficult to accurately capture cell subpopulations that significantly correlate with the therapeutic effects of MSCs. Recent evidence has demonstrated that an approach based on single-cell morphological profiling can be used for identifying functional cell subpopulations to predict clinical effectiveness of MSCs.^14,15^ Therefore, characterizing MSC heterogeneity at the single-cell level rather than in the bulk cell population would greatly contribute to the promotion of the clinical application and industrial development of MSCs in the future.

Proangiogenesis is considered effective in treating ischemic diseases such as limb ischemia, coronary heart disease, and ischemic stroke. Vascular endothelial growth factor (VEGF) is a potent and essential pro-angiogenic factor. Therefore, angiogenic therapy with VEGF is a clinically promising strategy for ischemic diseases.^16,17^ For example, MSCs have the potency to promote angiogenesis via paracrine effects from angiogenic factors such as VEGF.^18^ Various studies also have reported that MSCs are involved in angiogenesis and are useful in improving different ischemic diseases, such as hindlimb ischemia.^19^ However, due to the heterogeneity of MSCs, the detailed molecular mechanisms that optimize their angiogenic effect are not well understood.

In this study, as an indicator to assess angiogenic potential in MSCs, we established a robust in vitro method for the measurement of their ability to produce VEGF under ischemic conditions. Furthermore, using single-cell RNA-sequencing (scRNA-seq), which is currently used as one of the most powerful tools for understanding cellular heterogeneity, we highlighted the importance of elucidating functional cell subpopulations contributing to VEGF secretion ability under ischemic conditions. Our work could provide valuable information for further studies on identifying functional cell subpopulations or biomarkers to improve the efficiency of MSC-based product manufacturing and could help address the clinical challenges resulting from MSC heterogeneity.

## Materials and Methods

### Cell Cultures and Ischemic Treatment

Human bone marrow-derived MSCs (BM-MSCs) were obtained from LONZA (Walkersville, MD) (PT-2501), and ScienCell Research Laboratories (Carlsbad, CA) (7500). Human adipose-derived MSCs (AD-MSCs) were obtained from LONZA (PT-5006), ScienCell Research Laboratories (7510), and the American Type Culture Collection (ATCC, Manassas, VA) (PCS-500-011). Human umbilical cord-derived MSCs (UC-MSCs) were obtained from PromoCell (Heidelberg, Germany) (C-12971), ScienCell Research Laboratories (7530), and ATCC (PCS-500-010). All MSCs were maintained in MSCGM Mesenchymal Stem Cell Growth Medium BulletKit (LONZA) with penicillin/streptomycin under a humidified atmosphere of 5% CO_2_ and 95% air at 37 °C, according to the manufacturer’s instructions. The medium was changed every 2-3 days until 80%-90% confluency was reached. Thereafter, the cells were detached using trypsin/EDTA (LONZA) and reseeded for expansion and passaging. MSCs at passage 3/4 were frozen in STEM-CELLBANKER (Zenogen Pharma Co., Ltd, Fukushima, Japan) and cryopreserved in liquid nitrogen until use in further experiments.

**Figure 1.**
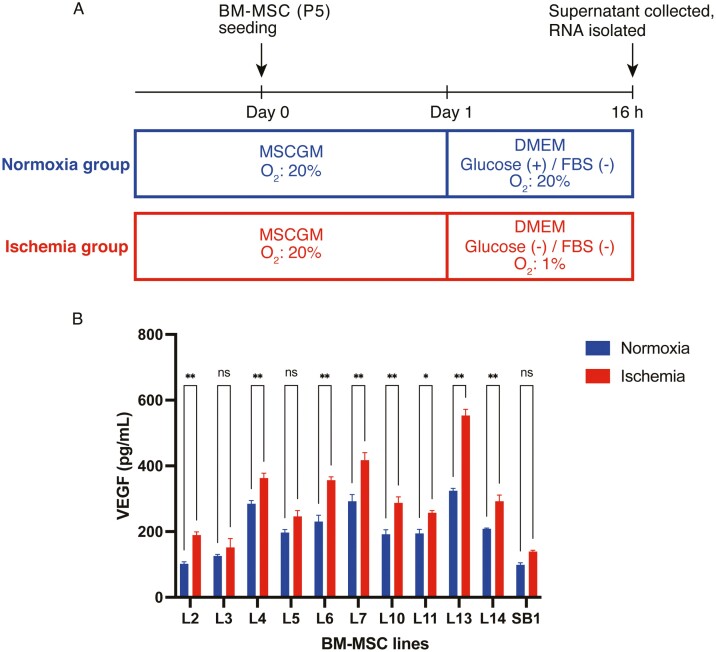
Effects of ischemia on VEGF secretion in 11 bone marrow (BM)-multipotent mesenchymal stromal/stem cells (MSCs) from different donors. (**A**) Schematic illustration of the protocol for culturing BM-MSCs under normoxic and ischemic conditions. (**B**) Variation in VEGF secretion among the 11 BM-MSC lines (L2, L3, L4, L5, L6, L7, L10, L11, L13, L14, and SB1) under normoxic and ischemic conditions. BM-MSCs were cultured under normoxic or ischemic conditions for 16 h. Level of VEGF secreted into the culture supernatant was determined using ELISA. The amount of VEGF secretion is given as the mean ± SEM (*n* = 5 for each group). *P < .05; **P < .01 (Student *t*-test).

To establish an in vitro cellular model to mimic ischemia, MSCs were cultured as previously described with some modifications.^20^ Briefly, MSCs at the passage number 5 were seeded into 24-well cell culture plates (3 × 10^4^ cells/well, Corning Inc., Corning, NY). After 24-h culture in MSCGM, the cells were incubated in Dulbecco’s modified Eagle’s medium (DMEM) without glucose and serum in the presence of penicillin/streptomycin in a hypoxic incubator (5% CO_2_, 1% O_2_ balanced with N_2_) for 16 h. As a normoxic control group, MSCs were cultured in high-glucose DMEM without serum in a standard incubator (5% CO_2_, 21% O_2_) for 16 h (Fig. 1A).

### Measurement of VEGF Secretion From MSCs

At 16 h after treatment as described above, the culture supernatant media were collected from both normoxic and ischemic treated groups, centrifuged at 2000 × *g* for 5 min at 4 °C to remove debris, and stored at −80 °C for VEGF-A measurement. Briefly, the supernatants from both normoxic and ischemic groups were added to the wells of a Human VEGF Quantikine ELISA plate (R&D Systems, Minneapolis, MN). Absorbance at 450 nm wavelength was measured using a microplate reader (ARVO X3, Perkin Elmer, Waltham, MA) according to the manufacturer’s instructions. A standard curve of known VEGF concentrations was generated to determine VEGF concentrations in unknown samples. Experiments were performed in at least triplicate.

### Single-Cell RNA-Seq Library Preparation and Sequencing

Single-cell libraries were prepared using Chromium NextGEM Single Cell 3ʹ Reagent Kits v3.1 (10X Genomics, Pleasanton, CA). The cells and kit reagents were mixed with gel beads containing the GEMs. The barcoded cDNAs in each GEM were pooled for PCR amplification, and adapter and sample indices were added after fragmentation targeting the 3ʹ end of the RNA. The generated libraries were paired-end sequenced on a HiSeq 2500 (Illumina, San Diego, CA) instrument, using the following read length: 28 bp Read1, 8 bp I7 index, and 91 bp Read2.

### Single-Cell Data Processing and Clustering

The analysis pipelines in Cell Ranger version 3.1.0 (10X Genomics) were used for data processing. FASTQ files were generated using cellranger mkfastq with the default parameters. Then, the cellranger count was run with –transcriptome = refdata-cellranger-GRCh38-3.0.0 for each sample, in which reads were mapped onto the human genome (GRCh38/hg38), and UMIs were counted for each gene. A clustering analysis was performed using the Seurat package (version 2.3.4). The UMI count for each sample was combined into a single Seurat object. Cells with fewer than 200 genes, more than 6000 genes, or more than 5% or 10% mitochondrial content were excluded as outliers. Model-based analysis of single-cell transcriptomics^21^ was performed to identify differentially expressed genes (DEGs) in each cluster/subpopulation to define MSC heterogeneity (*FindAllMarkers* (only.pos = TRUE, min.pct = 0.25, logfc.threshold = 0.25)). Then, Gene Ontology (GO) enrichment analysis was performed to analyze the functional roles of each cluster using *plot_all_cluster_go* function in the Scillus R package.

### Correlation Calculation and Scatter Plotting

The following calculation processes were performed in R (4.1.1). The cell number rate in each cluster relative to the total number of cells captured by scRNA-seq in each MSC line (lot) was calculated using the following formula:


$$\begin{array}{l} R_{ij}\left(\%\right)\;=\;\left(N_{ij}/NT_{j}\right)\;\times \;100 \end{array}$$

Here, *N*_*ij*_ is the cell number of cluster *i* in MSC lot#*j*, NT_*j*_ is total cell number in MSC lot#j, and *R*_*ij*_ is the cell number rate (percentage) of cluster *i* in MSC lot#*j*.

Using Spearman’s rank correlation (*r*_*s*_) method, the correlation coefficient between *R*_*ij*_ and the concentration of VEGF secretion in each MSC line cultured under ischemic conditions was calculated per cluster. Scatter plots were generated with the *ggscatter* function in the *ggpubr* package (version 0.5.0). A cluster showing a significant positive correlation (*P* < .05) was selected as the candidate cell subpopulation.

### Integrated Analysis of Multimodal Single-Cell Datasets

To find integration anchors between 2 scRNA-seq datasets (original scRNA-seq data [reference set] vs. test set), MSC data as the test set were merged using *FindTransferAnchors* function with MSC data as the reference set. Then, each cluster in the test set MSCs was classified based on the clusters obtained from the MSC data as a reference set using *TransferData* function.

### Small Interfering (si)RNA Transfection

For knockdown experiments using siRNA, MSCs were seeded in 6-well plates at a density of 9.5 × 10^4^ cells/well, and Silencer Select siRNAs were transfected at a final concentration of 25 pmol/well using Lipofectamine RNAiMAX Transfection Reagent (Thermo Fisher Scientific, Waltham, MA) according to the manufacturer’s instructions. The following Silencer Select siRNAs were used: *LRRC75A* (s52112, s52113), *CRYAB* (s3543), *HSPB1* (s6991), *FLG* (s5263), and negative control #1 (all purchased from Thermo Fisher Scientific). One day after siRNA transfection, the cells were washed twice with phosphate-buffered saline (PBS), and then 2 mL of new media for normoxic or ischemic treatments was added to each well. Culture supernatants were collected at 16 h after cultivation under normoxic or ischemic conditions to measure VEGF secretion from MSCs. Simultaneously, the cells were collected for total RNA extraction.

### Quantitative Real-Time PCR

Total RNA from MSCs was extracted using an RNeasy Mini QIAcube Kit (Qiagen, Hilden, Germany) on a QIAcube instrument (Qiagen) following the manufacturer’s instructions. RNA concentration was quantified using a NanoDrop One spectrophotometer (Thermo Fisher Scientific). Thereafter, total RNA was stored at −80 °C in single-use aliquots for later use. Real-time qPCR was performed using QuantiTect Probe RT-PCR Kit (Qiagen) on a StepOnePlus Real-Time PCR System instrument (Thermo Fisher Scientific) according to the manufacturer’s protocol. The expression levels of the selected genes were normalized to those of β-actin. The probes and primers used in this study were obtained from Sigma-Aldrich (St. Louis, MO) (listed in Supplementary Table S1).

### Statistical Analysis

All experiments were performed at least thrice. Values are shown as mean ± SE of the mean. For statistical comparisons, an unpaired Student’s *t*-test was performed to assess differences between groups or cell lines (eg, with or without ischemic treatment) using GraphPad Prism version 9 (GraphPad Software Inc., San Diego, CA). Statistical significance was set at *P* < .05.

## Results

### VEGF Secretion in Various BM-MSCs Under Ischemic Conditions

We first developed in vitro culture models of “ischemia” to investigate VEGF production in MSCs under ischemic environmental conditions. In most in vitro culture models of ischemia, a combination of oxygen and glucose deprivation in a culture environment mimics in vivo ischemic conditions.^22^ In addition, to prevent serum-derived VEGF from interfering with the quantification of MSC-derived VEGF in the culture medium, serum was removed in the current ischemic culture model. We obtained 11 BM-MSC line which were maintained in MSCGM^TM^ and displayed a fibroblast-like morphology (Supplementary Fig. S1A, S1B). To determine VEGF production under ischemic conditions in BM-MSCs, all the BM-MSC lines were maintained in MSCGM^TM^ until passage 5 (Fig. 1A). The amount of VEGF released in the culture supernatants was measured after 16 h incubation under normoxic and ischemic conditions. No obvious MSC cell death due to ischemia was observed. Also, VEGF expression was significantly enhanced under ischemic conditions in all the BM-MSC lines (Fig. 1B). Consistent with previous reports,^23-25^ the BM-MSC lines used in the current study also secreted VEGF in a low-oxygen environment. However, VEGF secretion under ischemic culture conditions differed among the 11 BM-MSC lines (Fig. 1B). Considering that all BM-MSC lines were cultured under the same conditions, these results suggest that the differences in VEGF secretion capacity after ischemia treatment may be attributable to differences in characteristics among the BM-MSC lines reflecting their gene expression profiles before ischemia.

### Characteristics and Single-Cell Transcriptome Profiles of BM-MSCs

**Figure 2.**
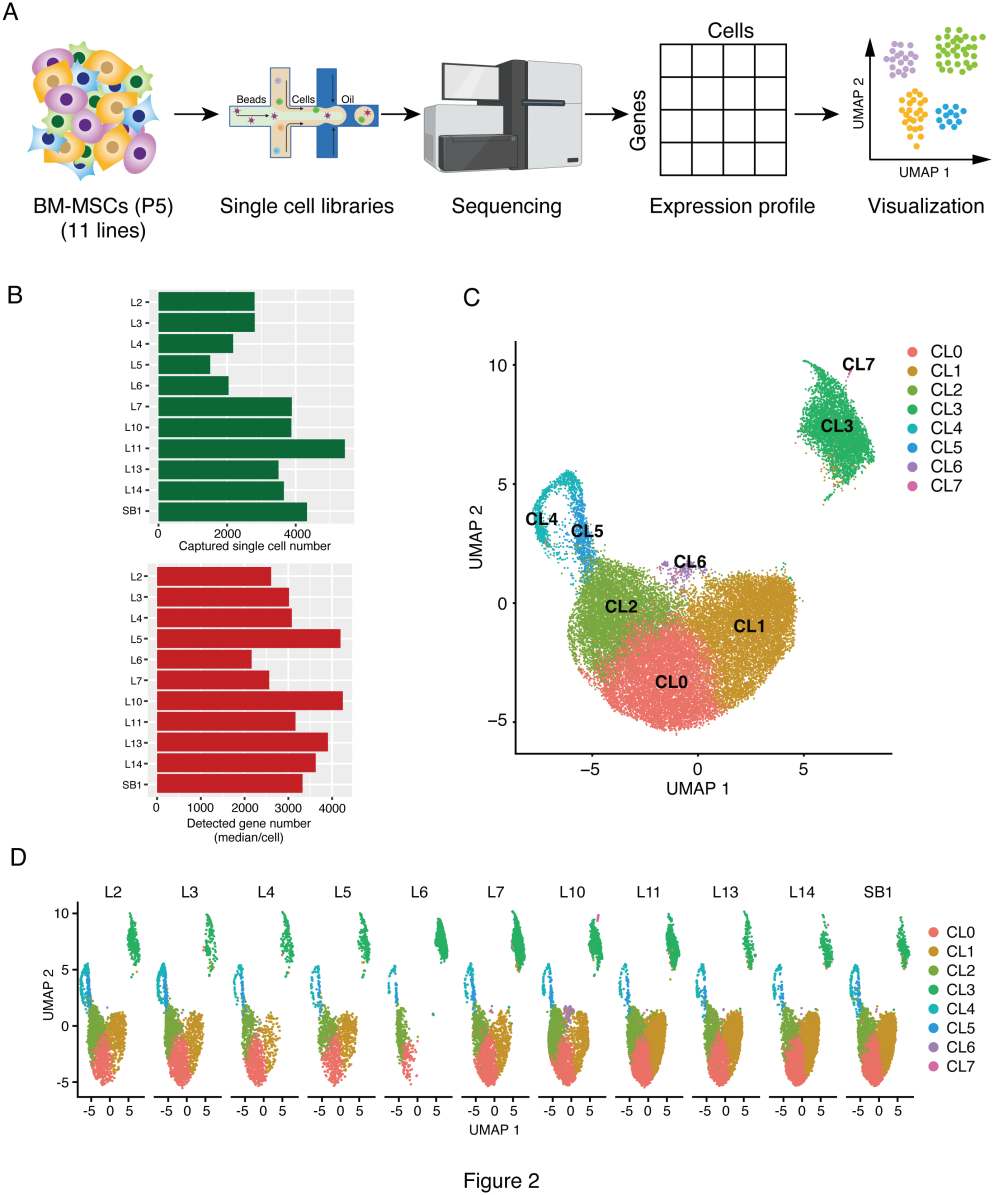
Single-cell transcriptome profiling of 11 bone marrow (BM)-multipotent mesenchymal stromal/stem cells (MSCs) lines from different donors. (**A**) Schematic illustration of single-cell transcriptome experiments. BM-MSCs under normoxic conditions were individually captured for barcoding using Chromium controller (10X Genomics), and barcoded cDNA libraries were sequenced using Illumina sequencer. Then, individual cells were grouped based on their transcriptome similarity through unsupervised clustering (UMAP). (**B**) Number of captured cells that passed quality control and the median number of detected genes per cell for each MSC line under normoxic conditions. (**C**) Visualization based on 2-dimensional UMAP plotted as UMAP1 (*x*-axis) vs. UMAP2 (*y*-axis) for each cell type of the 11 BM-MSC lines under normoxic conditions. Eight clusters (CL0-7) marked with different colors were obtained using Seurat’s graph-based clustering algorithm. (**D**) UMAP visualization for each of the 11 BM-MSC lines.

Next, we profiled the differences in VEGF secretion during ischemia among the BM-MSC lines (Fig. 1B). Because BM-MSCs are heterogeneous in nature,^26^ we hypothesized that different cell subsets express different levels of VEGF in the MSC population. To comprehensively examine gene expression in MSCs at the single-cell level, we performed droplet-based scRNA-seq (10X Genomics) on the 11 BM-MSC lines under normoxic conditions (Fig. 2A). The total number of cells recovered from all the BM-MSC lines using the 10X Genomics protocol was 36 001 (min: 1506 in L5, max: 5432 in L11, average: 3273), and the median number of genes per cell was 2161 (L6)–4243 (L10) (average: 3260), which satisfied our criteria (>200 and <6000 genes/cell) (Fig. 2B). Next, scRNA-seq data from all the BM-MSC lines were integrated, and the differences in heterogeneity among the BM-MSC lines were analyzed. Based on the mRNA expression profiles per cell in the 11 BM-MSC lines, we visualized the cells in two-dimensional space using uniform manifold approximation and projection (UMAP), a nonlinear dimension reduction method. Using the unsupervised k-means clustering algorithm, UMAP-assisted k-means clustering, we identified 8 distinct clusters (cell subpopulations) representing different cell types after integrating the 11 BM-MSC samples (Fig. 2C). In addition, the UMAP plot of each BM-MSC line revealed that all clusters were independent of each scRNA-seq library for the 11 BM-MSC lines, and no batch effect leading to local enrichment of a particular library was observed (Fig. 2D). Moreover, each BM-MSC line had 8 subpopulations, and the cell abundance of each subpopulation was different in each line (Fig. 2D).

To elucidate the functional characteristics of the subpopulations comprising BM-MSCs, marker genes were extracted for each cluster based on significant DEGs, and GO enrichment analysis was performed. The cells in clusters 0 and 1 (CL0 and CL1) were more likely to share GO terms related to cartilage and bone metabolism, such as “extracellular matrix organization” and “extracellular structure organization” (Supplementary Fig. S2), and may play essential roles in cartilage and bone tissue homeostasis. Cluster 2 (CL2) was enriched for platelet-like cells, such as “platelet degranulation” and “platelet activation” (Supplementary Fig. S2). These findings suggest that cells in CL0, CL1, and CL2 have characteristics related to the MSC multilineage differentiation potential. Cluster 3 (CL3) was enriched for GO terms related to mitochondrial metabolism, such as “hydrogen ion transmembrane transport” and “proton transport” (Supplementary Fig. S2), while the GO term “extracellular matrix/structure organization” was negatively regulated (Supplementary Fig. S2), suggesting that the cells in CL3 might be involved in migration to regenerate injured tissues. Cells in clusters 4 and 5 (CL4 and CL5) shared several GO terms related to cell division, such as “mitotic cell cycle” and “nuclear division,” suggesting that they are a cell population with high proliferative potential (Supplementary Fig. S2).

### BM-MSC Subpopulation Underlying VEGF Expression Capacity Under Ischemia

**Figure 3.**
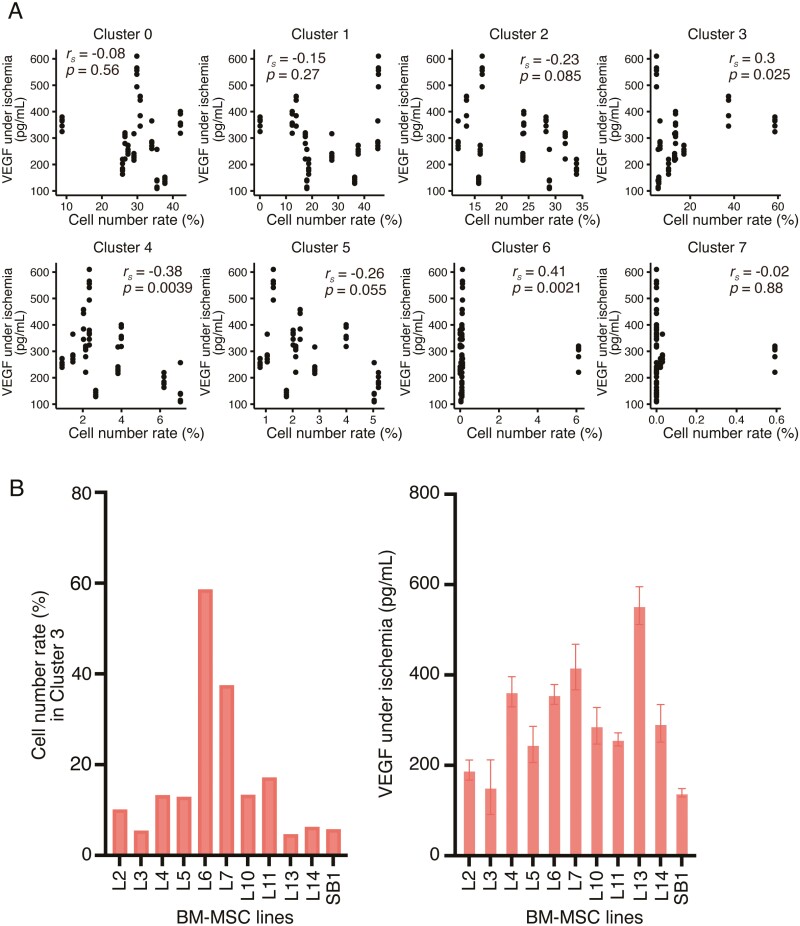
Correlation between cell number of each bone marrow (BM)-multipotent mesenchymal stromal/stem cell (MSC) line in each cluster obtained from scRNA-seq and VEGF secretion level in each BM-MSC line under ischemic conditions. (**A**) Scatter plots and Spearman’s rank correlation coefficient between the rank of cell number of each BM-MSC line in each cluster and the rank of VEGF secretion level in each BM-MSC line under ischemic conditions. The *x*-axis represents the percentage of the number of cells in each cluster relative to the total number of cells captured by scRNA-seq in each cell line. The *y*-axis represents VEGF secretion in each cell line under ischemic conditions. The positive correlation of CL3 (*r*_*s*_ = 0.3) as determined using Spearman’s rank correlation is shown. In the case of *n* = 55 data points, the observed value of *r*_*s*_ must be more than 0.266 (positively correlated) to be considered significant (*P*-value < .05). *r*_*s*_, Spearman’s rank correlation coefficient. (**B**) Bar graphs presenting the cell number of each BM-MSC line in CL3, which showed a positive correlation in (**A**) (left graph) and the VEGF secretion levels in 11 BM-MSC lines under ischemic conditions (right graph; Data are extracted from Fig. 1B).

Next, we attempted to identify the candidate cell subpopulation that correlates with VEGF-producing ability under ischemic conditions from the clusters shown in Fig. 2C. We calculated Spearman’s rank correlation between the number rate of cells in each BM-MSC line in each cluster and the VEGF secretion level in each BM-MSC line under ischemic conditions. Only in CL3, we observed a significant positive correlation (Spearman’s rank correlation, *r*_*s*_ = 0.3, *P* < .05) between the cell number rate of each BM-MSC line and VEGF secretion ability under ischemic conditions (Fig. 3A, 3B). Next, when we investigated the gene expression profile of CL3, the most upregulated gene in CL3 was *LRRC75A* (Table 1), compared with the other clusters. In contrast, the downregulated genes in CL3 included *NEAT1*, *FOS*, *COL3A1*, *ZFP36L1*, *LEPR*, *PLPP3*, and *COL6A3* (Supplementary Fig. S3). Notably, although the differences were less than 1.5-fold, compared with the other clusters, the CL3 subpopulation also showed higher base-level expression of VEGF family genes (*VEGF-A*, *VEGF-B*, and *VEGF-C*) (Supplementary Fig. S4A–S4C).

**Table 1.** Top 20 upregulated DEGs of cluster 3.

Gene name	Ave log_2_FC	*P* value
*LRRC75A*	1.0357	0
*KRT7*	0.8382	0
*KRT16*	0.7902	2e−291
*C1orf56*	0.7815	0
*CRYAB*	0.7696	0
*HSPB1*	0.7572	0
*MTRNR2L12*	0.7060	0
*AC092069.1*	0.7024	0
*ADIRF*	0.6712	0
*LGALS1*	0.6573	0
*ID1*	0.6525	0
*MT2A*	0.6424	0
*S100A11*	0.6312	0
*COMP*	0.6132	1e−312
*EIF5A*	0.6057	0
*FLG*	0.6049	8e−90
*SH3BGRL3*	0.5970	0
*TPM2*	0.5859	0
*POLR2L*	0.5555	0
*GADD45B*	0.5543	0

Abbreviations: Ave Log_2_FC, average log2 fold-change; DEGs, differentially expressed genes.

Next, we examined whether CL3 could be similarly identified in the 4 other BM-MSC lines (L15–18) prepared independently as a test set. scRNA-seq data from the 4 BM-MSC lines for the test set showed that their subpopulations were classified into 8 clusters, confirming that the test set BM-MSCs (L15–18) were also composed of a heterogeneous cell population, as well as the results of the original scRNA-seq (L2-SB1) (Supplementary Fig. S5A). To investigate how the individual cells in the test set (L15–18) were categorized as cell types classified by scRNA-seq (L2-SB1), an integrative analysis of the scRNA-seq (L2-SB1), and the test set scRNA-seq (L15-L18) was performed using scRNA-seq data (L2-SB1) as a reference set (Supplementary Fig. S5A). Cluster 7 (TS7) in the test set was mainly transferred to CL3 in the reference set (Supplementary Fig. S5B). To estimate whether the cells in the test set classified as CL3 correlated with the production ability of VEGF during ischemic conditions, we calculated the Spearman’s rank correlation coefficient between the cell number rate of each BM-MSC line in test set CL3 and VEGF secretion levels in its BM-MSC line under ischemic conditions. The number of cells in the cell line classified as CL3 using integrative analysis was significantly correlated with the ability to produce VEGF during ischemic conditions (Spearman’s rank correlation, *r*_*s*_ = 0.63, *P* < .01) (Supplementary Fig. S5C).

Furthermore, we analyzed other sources of adipose-derived MSCs (AD-MSCs) as cells for another test set for the presence of the CL3 subpopulation. Four different donor-derived AD-MSC lines were obtained from several suppliers. Unsupervised clustering of mRNA expression data obtained from all cells in the 4 AD-MSC lines under normoxic conditions revealed 11 clusters (Supplementary Fig. S6A). To determine where CL3 is classified on UMAP for AD-MSCs, AD-MSC scRNA-seq data were used for an integrated analysis with the reference set scRNA-seq data (L2-SB1). Cluster 0 (AD0) in AD-MSC scRNA-seq was mainly transferred to CL3 in the reference set (Supplementary Fig. S6B). Additionally, we estimated whether the AD-MSC subpopulation classified as CL3 correlated with the ability to produce VEGF under ischemic conditions. Similar to the case of the BM-MSC test set (L15–18), we observed a high correlation (Spearman’s rank correlation, *r*_*s*_ = 0.51, *P* < .05) between the cell number rate for each AD-MSC line in subpopulation classified as CL3 and the ability to produce VEGF across AD-MSC lines under ischemic conditions (Supplementary Fig. S6C). These results strongly suggest that the presence of a subpopulation classified as CL3 correlates with the ischemic VEGF secretion level of MSCs.

### *LRRC75A* is a Regulator for VEGF Secretion in BM-MSCs and AD-MSCs Under Ischemia

**Figure 4.**
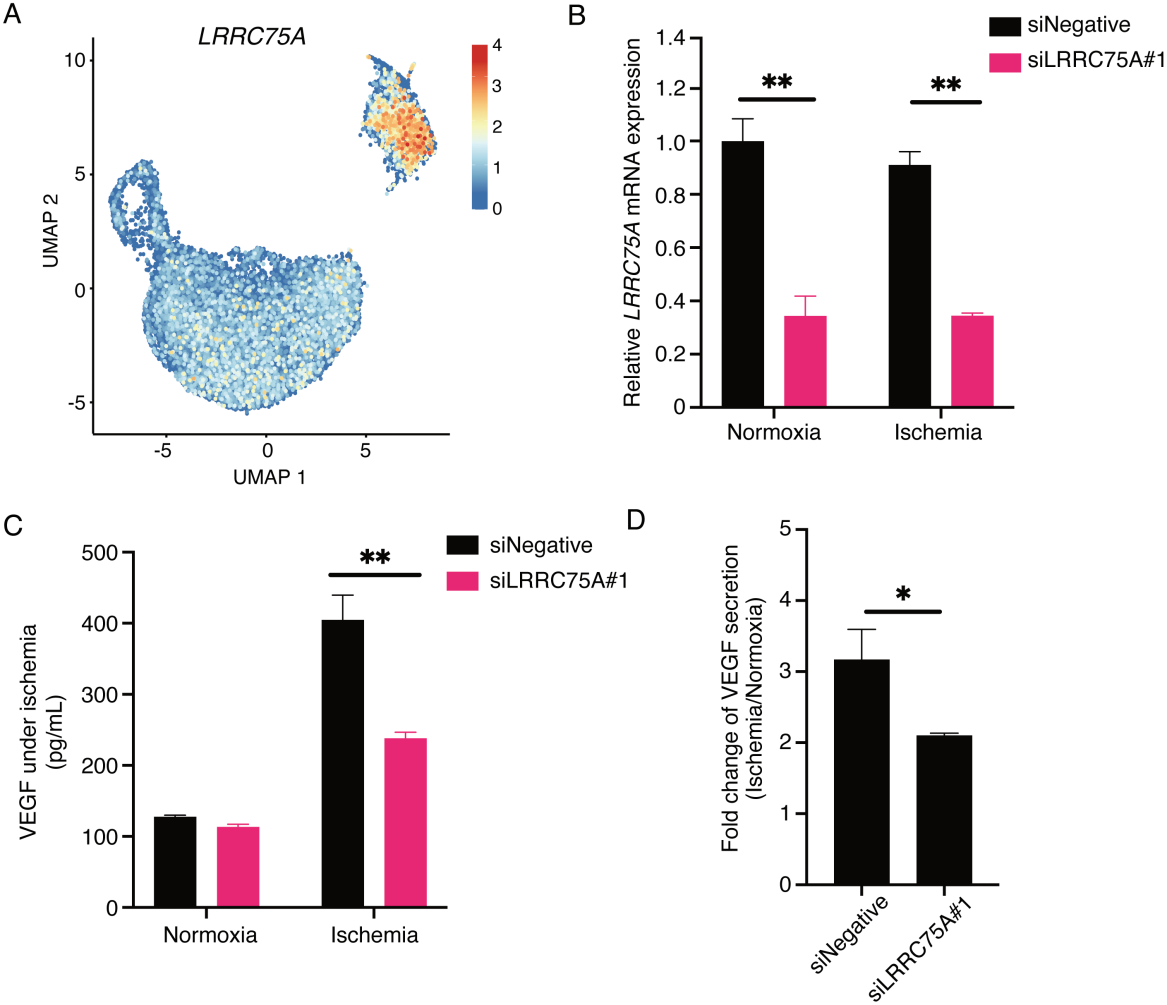
Silencing of *LRRC75A* expression affects VEGF secretion induced by ischemic treatment. (**A**) Expression of *LRRC75A* is represented using UMAP visualization (cell positions are from the UMAP plot in Fig. 2C). *LRRC75A* was more highly expressed in CL3 than in other clusters. (**B and C**) Bone marrow (BM)-multipotent mesenchymal stromal/stem cells (MSCs) were transfected with either control siRNA (siNegative) or *LRRC75A* siRNA (siLRRC75A#1) under normoxic and ischemic conditions. mRNA level for *LRRC75A* (B) and VEGF secretion level in the supernatant (C) were determined using qPCR and ELISA, respectively. (**D**) Fold change of VEGF secretion level under ischemic conditions relative to that under the normoxic conditions are represented based on the result shown in (C). All values indicate the mean ± SEM of 3 independent biological replicates. (*P < .05, **P < .01, Student’s *t*-test).

To molecularly characterize the mechanism that regulates VEGF secretion in CL3, we focused on genes that were the most differentially expressed in CL3. *LRRC75A* was more strongly expressed in CL3 than in other clusters (Table 1; Figure 4A). Notably, the test sets of BM-MSCs and AD-MSCs also exhibited high *LRRC75A* expression in their matched CL3 clusters (Supplementary Fig. S4D and S5D). To investigate whether *LRRC75A* regulates VEGF secretion under ischemic conditions, a loss-of-function assay using *LRRC75A* siRNA was performed in normoxic and ischemic BM-MSCs. The knockdown efficiency for *LRRC75A* in BM-MSCs was confirmed by analyzing the expression of *LRRC75A* (Fig. 4B). Knockdown of *LRRC75A* effectively suppressed VEGF secretion in BM-MSCs (Fig. 4C). In particular, VEGF production under ischemic conditions was markedly suppressed by *LRRC75A* knockdown compared with functional loss of *LRRC75A* under normoxic conditions (Fig. 4D). Similarly, *LRRC75A* knockdown in AD-MSCs also revealed that decreased *LRRC75A* expression effectively downregulated VEGF secretion in AD-MSCs under ischemic conditions (Supplementary Fig. S7A, S7B). Furthermore, to verify that off-target effects did not induce this suppression, we estimated the VEGF suppressive effect using different designed siRNAs targeting *LRRC75A*. Another *LRRC75A* siRNA effectively suppressed VEGF secretion under ischemic conditions (Supplementary Fig. S8A, S8B). On the contrary, no significant suppressive effect on VEGF expression was observed (Supplementary Fig. S9A–S9F) when ­loss-of-function experiments were performed with siRNAs targeting other genes that were enriched in CL3 (eg, *CRYAB*, *HSPB1*, *FLG*, selected from Table 1). Our data further showed that in UC-MSCs that secreted a lower level of VEGF even under ischemic conditions, there were significantly fewer *LRRC75A*-expressing cells in the CL3 subpopulation (Supplementary Fig. S10A–S10D). Therefore, the *LRRC75A*-mediated pathway may be involved in the induction of VEGF expression during ischemia in BM-MSCs and AD-MSCs.

## Discussion

To quantify the angiogenic potential of BM-MSC lines, we developed a robust in vitro culture method to assess the VEGF production of MSC lines under ischemic culture conditions (1% oxygen, no glucose, no serum) with low inter-experimental variability. In addition, we elucidated the heterogeneity of BM-MSCs using unsupervised clustering of scRNA-seq data among 11 donor-different BM-MSC lines and identified 8 cell subpopulations. These observed cell subpopulations suggest varying functional cellular states in BM-MSCs.

GO analysis results revealed that the cell subpopulations categorized as CL0 and CL1 were strongly related to pathways that contribute to cartilage and bone tissue homeostasis. Furthermore, this observation is consistent with those from previous studies that showed that MSCs can differentiate into osteogenic or chondrogenic cells.^27,28^ Therefore, CL0 and CL1 might be MSC subpopulations with a differentiation bias for osteocytes or chondrocytes. In addition, GO analysis revealed that CL2 might possess characteristics similar to platelet-like cells.^29,30^ These findings suggest that CL0, CL1, and CL2 could affect the multilineage differentiation potential of BM-MSCs. CL3 was annotated as a cell subpopulation with enhanced mitochondrial functions. Cellular senescence and mitochondrial dysfunction are both typical features of the aging process.^31^ Therefore, high mitochondrial function is crucial for the maintenance of essential cellular functions. Our GO analysis also revealed that extracellular matrix-related genes were downregulated in CL3, suggesting that the reduced cell–cell adhesive potential due to the decrease in the surrounding extracellular matrix causes the cells to be more migratory. Cell migration is one of the various cellular processes that require mitochondrial energy.^32^ Therefore, the ­mitochondrial-gene-enriched CL3 might be a cell subpopulation that exhibits migratory potential to the cell. BM-MSCs also contained cell subpopulations annotated as proliferative stem-like cells (CL4 and CL5). BM-MSCs are multipotent and possess a potential for trilineage differentiation toward osteogenic, chondrogenic, and adipogenic fates.^33^ Accordingly, it is suggested that the proliferative stem-like cells in CL4 and CL5 are in a cell lineage-priming state, allowing cell differentiation toward several lineages. The number of cells classified as CL6 or CL7 was relatively small (Fig. 3A) and they were almost exclusively detected in one particular BM-MSC line (Fig. 2D). Thus, these 2 clusters are not really associated with VEGF secretion in the ischemic condition, though the *P*-value of CL6 was less than 1%.

LRRC75A is a leucine-rich repeat-containing (LRRC) protein belonging to the LRRC superfamily; however, little is known about its role. The LRRC superfamily comprises several 100 proteins characterized by 2 or more LRR motif proteins. LRRC proteins are also involved in supporting diverse cellular functions.^34,35^ In a recent report, scRNA-seq analysis showed that an *LRRC75A*-positive subpopulation in UC-MSCs was rich in gene transcripts associated with extracellular matrix organization, extracellular structure organization and ossification, suggesting that LRRC75A might be a potential novel osteogenic marker.^36^ In contrast, the *LRRC75A*-expressing subpopulation we discovered was characterized by extremely low expression of extracellular matrix-related genes compared to other subpopulations, and the GO analysis detected no clear correlation with osteogenesis. This may be due to the difference in the tissue of origin of MSCs between the 2 studies. Consistent with a previous study,^37^ we also showed that UC-MSCs secreted a lower level of VEGF than BM-MSCs. Moreover, the number of cells expressing *LRRC75A* in CL3 in UC-MSCs was significantly lower than in BM-MSCs and AD-MSCs, suggesting that the expression level of *LRRC75A* in the CL3 subset is a determinant of VEGF secretion potential in various tissue-derived MSCs. Some LRRC proteins activate NF-κB signaling to induce the expression of proinflammatory cytokines.^38,39^ It is also well-known that the expression of VEGF is regulated via the NF-κB pathway,^40,41^ suggesting that LRRC proteins contribute to VEGF expression via the NF-κB pathway. However, whether the intrinsic function of LRRC proteins in promoting VEGF secretion under ischemic conditions directly affects the signaling pathway induced by ischemic stress remains unclear. Notably, our experimental data showed for the first time that *LRRC75A* markedly upregulates VEGF secretion under ischemic stress. Therefore, our findings suggest that the VEGF pathway *via LRRC75A* is involved in suppressing the deterioration of angiogenesis during ischemic stress.

We inferred that CL3 might be a candidate marker for predicting angiogenic potency in BM-MSCs. Similar to our study, an FDA research group also reported other techniques for identifying functional subpopulations to predict the therapeutic effect of MSC-based products.^14,15^ Their strategy is based on live-phase morphological profiling of single MSCs. Therefore, MSC function can be predicted non-destructively by monitoring morphological differences during their manufacturing processes. In contrast, the scRNA-seq approach requires cell destruction; however, it helps to elucidate the molecular mechanisms underlying the therapeutic effects of MSCs. Indeed, we presented the first evidence that *LRRC75A* positively regulates VEGF expression under ischemic conditions through further studies of DEGs in CL3, indicating that *LRRC75A* and its product are potential biomarkers for predicting the angiogenic effects of BM-MSCs.

Lastly, we were unable to detect *LRRC75A* as a gene associated with VEGF secretion under ischemic conditions using bulk RNA-seq analysis (data not shown). Unlike scRNA-seq, gene expression levels obtained using bulk RNA-seq are provided as averages of all cells. Therefore, selective gene expression for a specific cell subpopulation, such as *LRRC75A*, could be undetectable using bulk RNA-seq. Hence, scRNA-seq analysis is a powerful tool for identifying genes expressed in rare cell types from heterogeneous MSCs since cellular heterogeneity is not masked. It would be methodologically challenging to identify key molecules in a specific functional cell subpopulation using either bulk RNA-seq or morphological profiling, suggesting that scRNA-seq is a useful analytical tool for identifying biomarkers and functional cell populations linked to pharmacological effects in heterogeneous MSCs. The current study increases our understanding of the heterogeneity associated with MSC function and may facilitate the identification of critical quality attributes and the setting of specifications for MSC-based therapeutic products as well as the validation of their manufacturing processes.

## Conclusion

We demonstrate the use of single-cell transcriptome profiling to identify the cell subpopulations involved in the ischemic VEGF-producing ability of BM-MSCs derived from multiple donors. Moreover, we revealed that *LRRC75A* might be a candidate biomarker for predicting the efficacy of angiogenic therapy with BM-MSCs and leverage the VEGF production of BM-MSCs at ischemic sites, which is considered one of the major mechanisms for MSC-induced angiogenesis. Hence, identifying cell subpopulations and biomarkers that correlate with therapeutic efficacy based on single-cell transcriptome profiles will help ensure the function of MSCs and contribute to the reproducible manufacturing of effective MSC-based therapeutic products.

## Supplementary Material

szad029_suppl_Supplementary_Material

## Data Availability

All raw scRNA-seq data analyzed in this study are available on GenBank (http://www.ncbi.nlm.nih.gov/Genbank/index.html), EMBL (http://www.ebi.ac.uk) and DNA Data Bank of Japan (http://www.ddbj.nig.ac.jp) under the accession numbers DRA015508 and DRA016064.
