# Supplementary material for: Single-Cell RNA-Seq Reveals LRRC75A-Expressing Cell Population Involved in VEGF Secretion of Multipotent Mesenchymal Stromal/Stem Cells Under Ischemia
Source: Stem Cells Transl Med. 2023 Jun 2;12(6):379–90. doi: 10.1093/stcltm/szad029 (PMC10267575; doi:10.1093/stcltm/szad029)
Supplement: szad029_suppl_Supplementary_Material [file szad029_suppl_supplementary_material.pdf]

Supplementary information for

Single-cell RNA-seq reveals *LRRC75A*-expressing cell population involved in VEGF secretion of multipotent mesenchymal stromal/stem cells under ischemia

Takumi Miura, Tsukasa Kouno, Megumi Takano, Takuya Kuroda, Yumiko Yamamoto, Shinji Kusakawa, Masaki Suimye Morioka, Tohru Sugawara, Takamasa Hirai, Satoshi Yasuda, Rumi Sawada, Satoko Matsuyama, Hideya Kawaji, Takeya Kasukawa, Masayoshi Itoh, Akifumi Matsuyama, Jay W Shin, Akihiro Umezawa, Jun Kawai, Yoji Sato

A

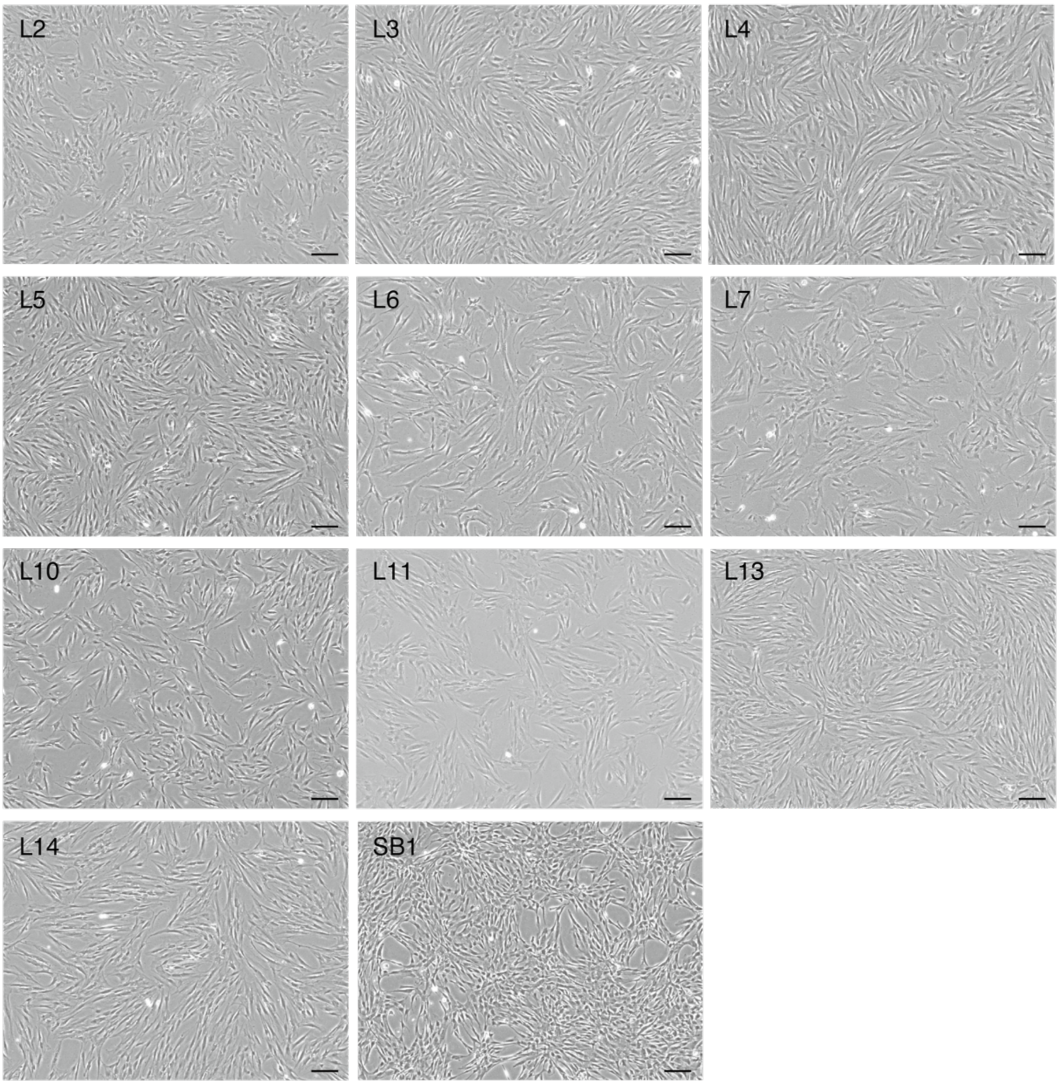

B

Cell ID	Lot. #	Supplier	Origin	Donor age (Y)	Sex
L2	18TL241909	Lonza	Bone marrow	36	Male
L3	18TL262066	Lonza	Bone marrow	25	Male
L4	18TL282222	Lonza	Bone marrow	26	Male
L5	18TL312488	Lonza	Bone marrow	24	Female
L6	19TL029340	Lonza	Bone marrow	24	Male
L7	19TL058658	Lonza	Bone marrow	23	Female
L10	19TL191055	Lonza	Bone marrow	19	Female
L11	19TL155677	Lonza	Bone marrow	31	Male
L13	19TL281098	Lonza	Bone marrow	25	Male
L14	19TL337479	Lonza	Bone marrow	37	Male
SB1	21580	ScienCell	Bone marrow	Unknown	Unknown

Supplementary Figure S1

Characteristics of bone marrow (BM)-multipotent mesenchymal stromal/stem cells (MSCs) in this study.

(A) Cell of 11 BM-MSC lines (L2, L3, L4, L5, L6, L7, L10, L11, L13, L14, and SB1) were spindle-shaped and resembled fibroblasts (scale bar = 200 μm).

(B) Table summarizing BM-MSC lot information. The cell ID was labeled for this study. Donor information (lot #, origin, age, and sex) is stated in the Certificate of Analysis from suppliers.

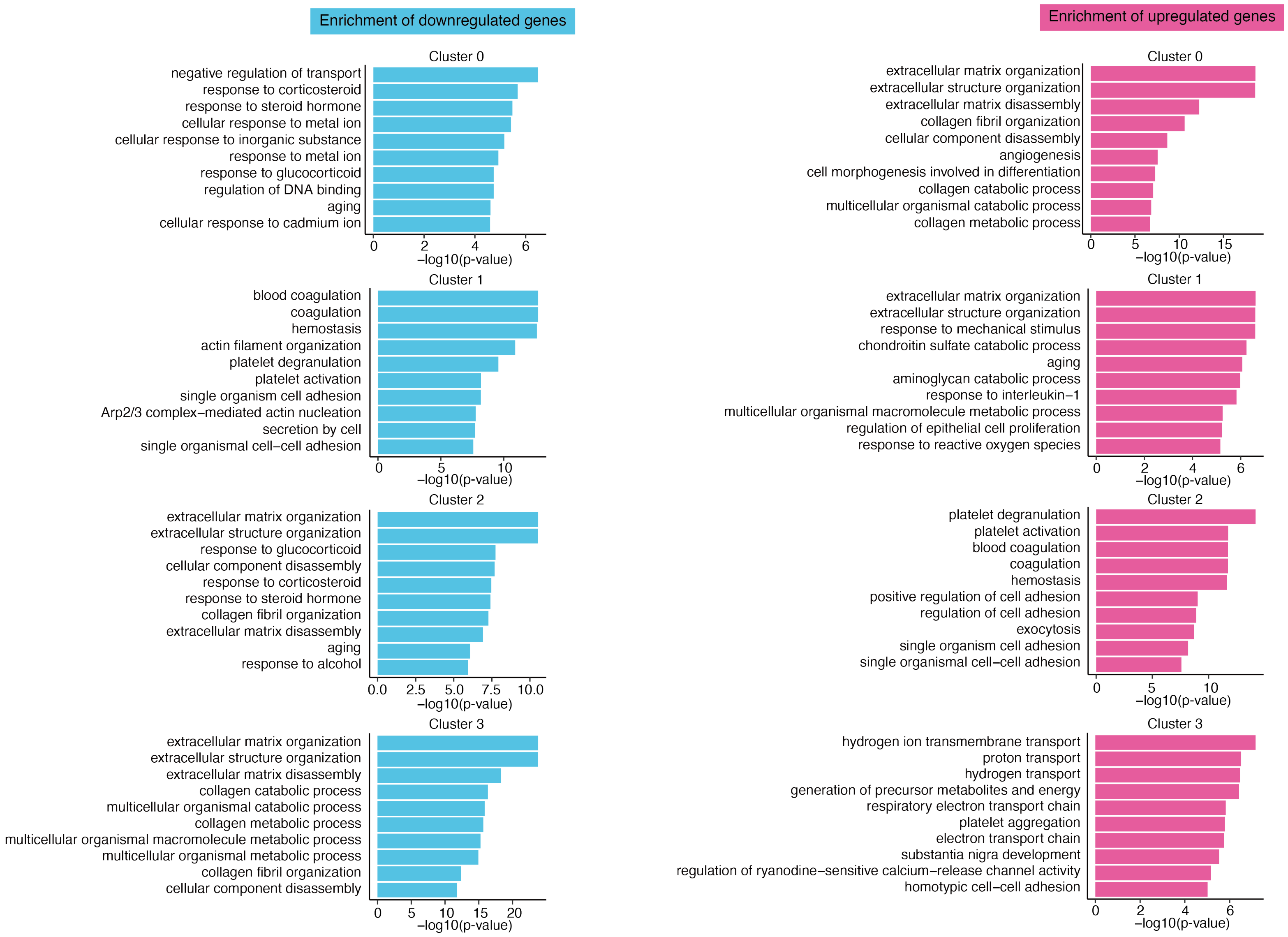

Supplementary Figure S2 continued on next page

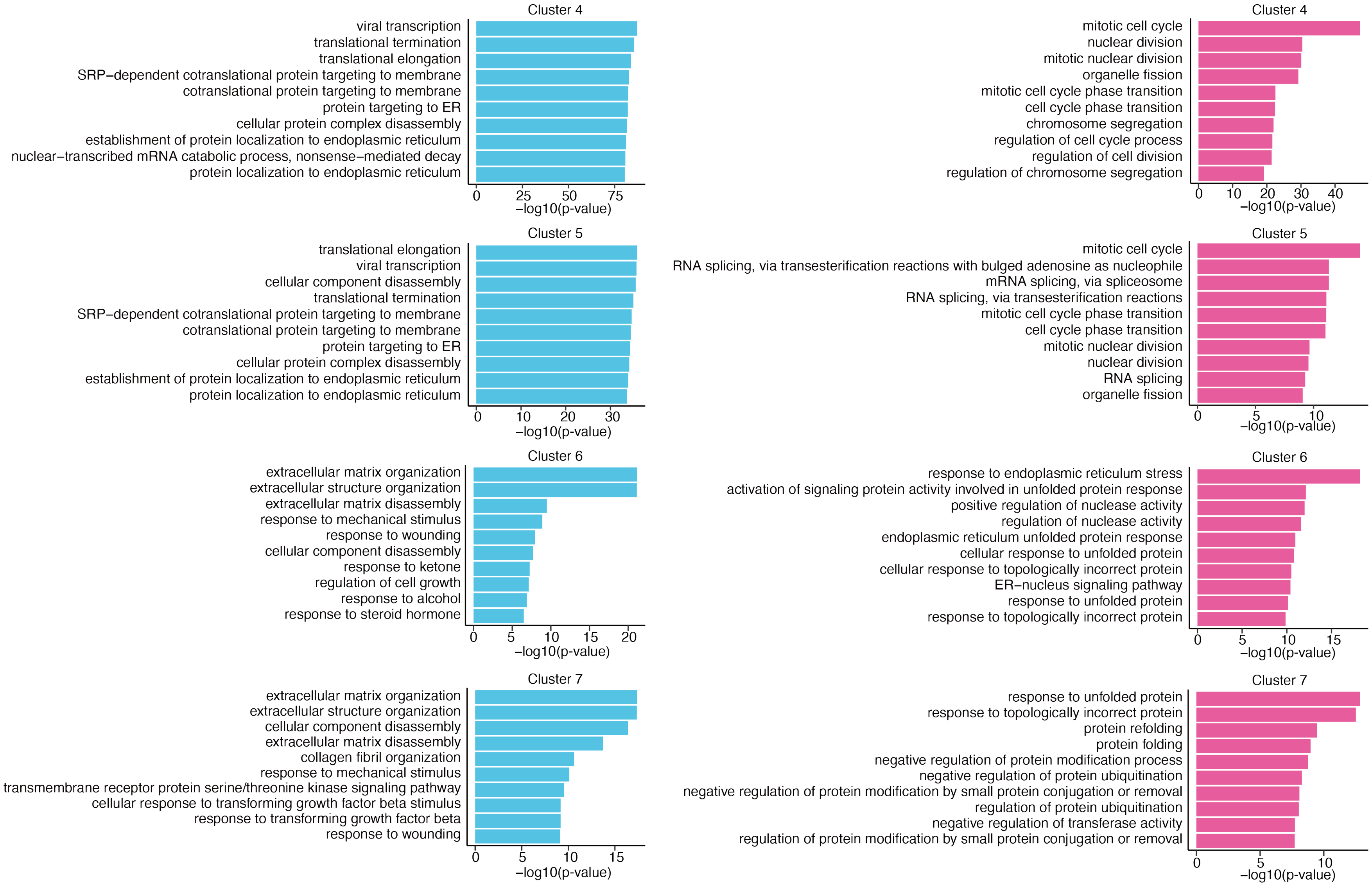
Cluster 4
viral transcription
translational termination
translational elongation
SRP−dependent cotranslational protein targeting to membrane
cotranslational protein targeting to membrane
protein targeting to ER
cellular protein complex disassembly
establishment of protein localization to endoplasmic reticulum
nuclear−transcribed mRNA catabolic process, nonsense−mediated decay
protein localization to endoplasmic reticulum
0 25 50 75
−log10(p-value)
Cluster 4
mitotic cell cycle
nuclear division
mitotic nuclear division
organelle fission
mitotic cell cycle phase transition
cell cycle phase transition
chromosome segregation
regulation of cell cycle process
regulation of cell division
regulation of chromosome segregation
0 10 20 30 40
−log10(p-value)
Cluster 5
translational elongation
viral transcription
cellular component disassembly
translational termination
SRP−dependent cotranslational protein targeting to membrane
cotranslational protein targeting to membrane
protein targeting to ER
cellular protein complex disassembly
establishment of protein localization to endoplasmic reticulum
protein localization to endoplasmic reticulum
0 10 20 30
−log10(p-value)
Cluster 5
mitotic cell cycle
RNA splicing, via transesterification reactions with bulged adenosine as nucleophile
mRNA splicing, via spliceosome
RNA splicing, via transesterification reactions
mitotic cell cycle phase transition
cell cycle phase transition
mitotic nuclear division
nuclear division
RNA splicing
organelle fission
0 5 10
−log10(p-value)
Cluster 6
extracellular matrix organization
extracellular structure organization
extracellular matrix disassembly
response to mechanical stimulus
response to wounding
cellular component disassembly
response to ketone
regulation of cell growth
response to alcohol
response to steroid hormone
0 5 10 15 20
−log10(p-value)
Cluster 6
response to endoplasmic reticulum stress
activation of signaling protein activity involved in unfolded protein response
positive regulation of nuclease activity
regulation of nuclease activity
endoplasmic reticulum unfolded protein response
cellular response to unfolded protein
cellular response to topologically incorrect protein
ER−nucleus signaling pathway
response to unfolded protein
response to topologically incorrect protein
0 5 10 15
−log10(p-value)
Cluster 7
extracellular matrix organization
extracellular structure organization
cellular component disassembly
extracellular matrix disassembly
collagen fibril organization
response to mechanical stimulus
transmembrane receptor protein serine/threonine kinase signaling pathway
cellular response to transforming growth factor beta stimulus
response to transforming growth factor beta
response to wounding
0 5 10 15
−log10(p-value)
Cluster 7
response to unfolded protein
response to topologically incorrect protein
protein refolding
protein folding
negative regulation of protein modification process
negative regulation of protein ubiquitination
negative regulation of protein modification by small protein conjugation or removal
regulation of protein ubiquitination
negative regulation of transferase activity
regulation of protein modification by small protein conjugation or removal
0 5 10
−log10(p-value)

Supplementary Figure S2

Gene Ontology (GO) analysis of differentially expressed genes (DEGs) in clusters classified by scRNA-seq (performed in Figure 2C). Red and blue bars indicate upregulated and downregulated genes, respectively.

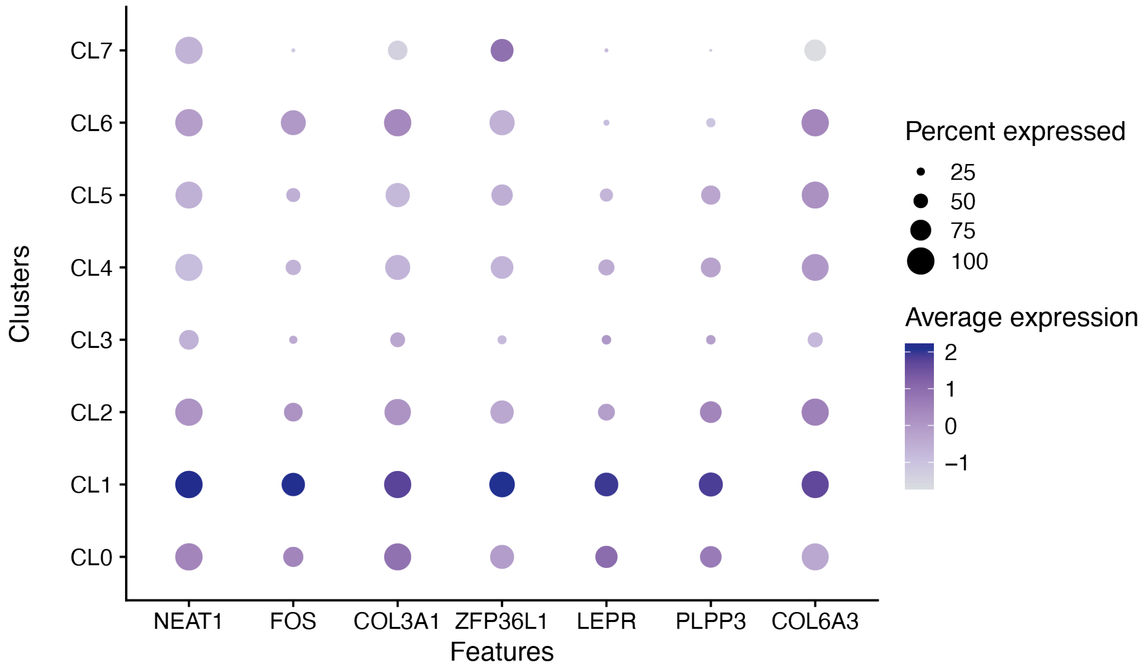

Supplementary Figure S3

Dot plot of expression of genes downregulated in CL3.

Gradient-colored dot plots indicate the average expression of a gene (low to high, shown as light to dark), and dot sizes indicate percentages of cells within each cluster expressing that gene.

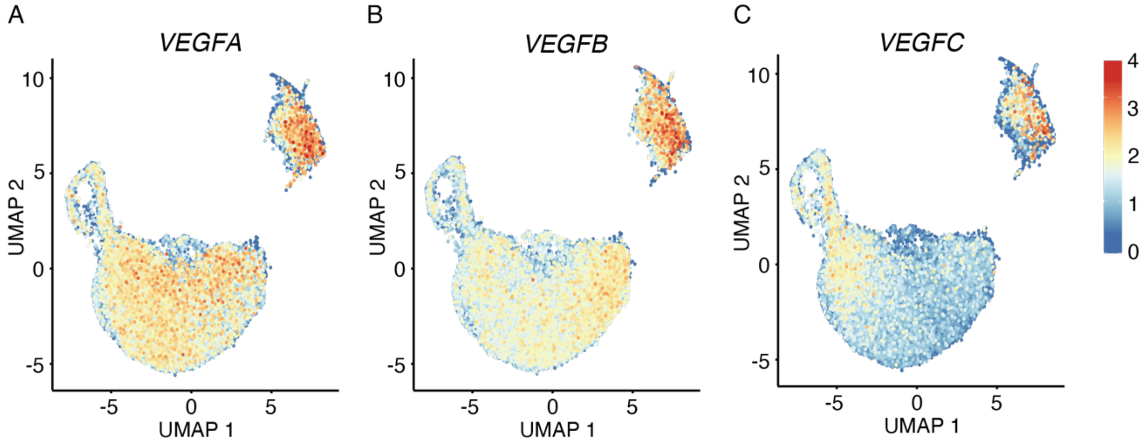

Supplementary Figure S4

Expression levels of *VEGF-A* (A), *VEGF-B* (B), and *VEGF-C* (C) genes were visualized using FeaturePlot (cell positions are from the UMAP plot in Figure 2C). These VEGF family genes were more expressed in CL3 than in other clusters (low to high, shown as a gradient from blue to red).

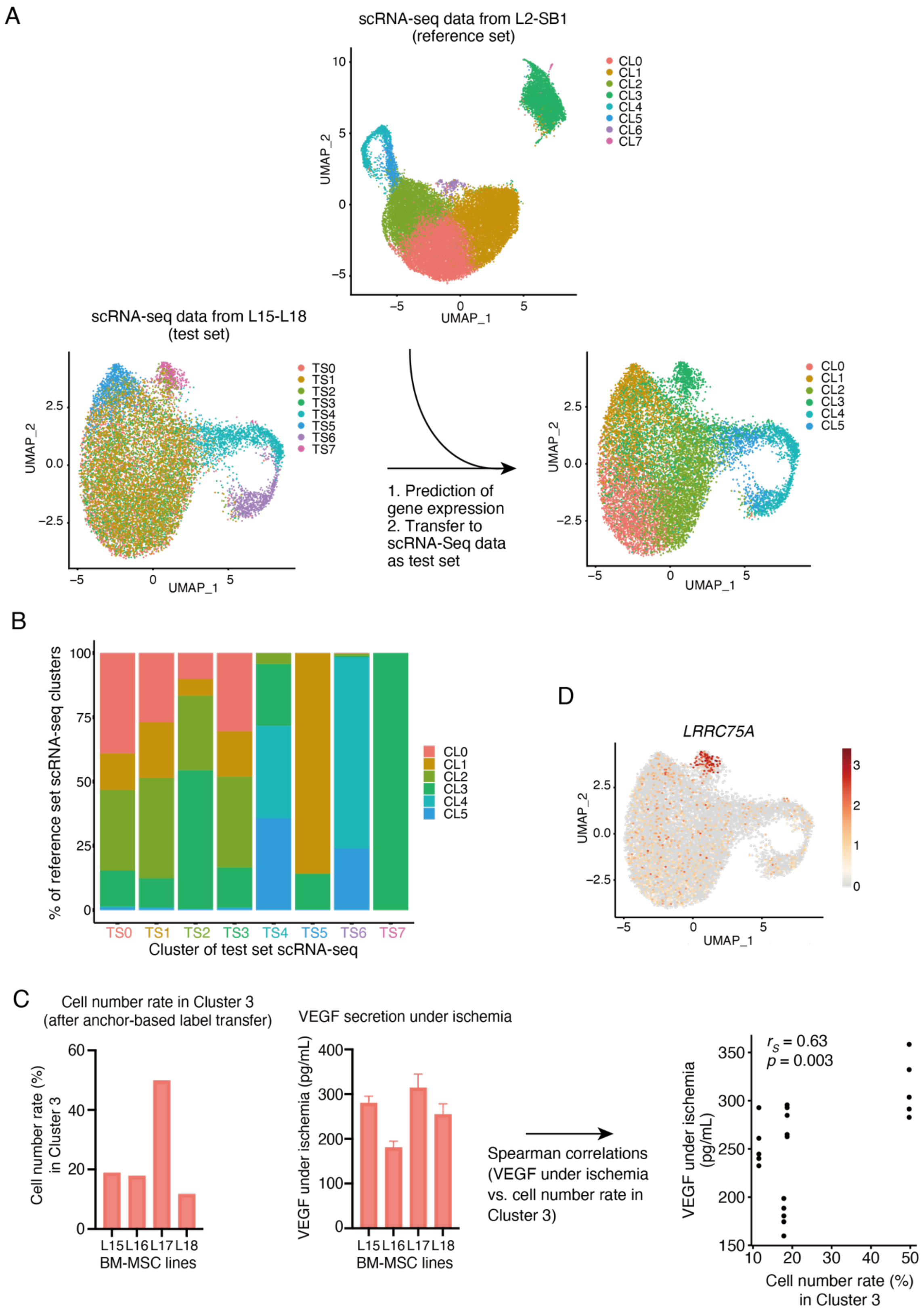
A
scRNA-seq data from L2-SB1
(reference set)
UMAP_1
UMAP_2
CL0
CL1
CL2
CL3
CL4
CL5
CL6
CL7
scRNA-seq data from L15-L18
(test set)
TS0
TS1
TS2
TS3
TS4
TS5
TS6
TS7
1. Prediction of gene expression
2. Transfer to scRNA-Seq data as test set
B
% of reference set scRNA-seq clusters
Cluster of test set scRNA-seq
C
Cell number rate in Cluster 3
(after anchor-based label transfer)
Cell number rate (%) in Cluster 3
BM-MSC lines
VEGF secretion under ischemia
VEGF under ischemia (pg/mL)
Spearman correlations (VEGF under ischemia vs. cell number rate in Cluster 3)
r_s = 0.63
p = 0.003
VEGF under ischemia (pg/mL)
Cell number rate (%) in Cluster 3
D
LRRC75A

Supplementary Figure S5

Identification of CL3 in another scRNA-seq dataset (different from the original scRNA-seq dataset in this study)

(A) Integrative analysis of reference set scRNA-seq data and test set scRNA-seq data. To identify cell types in test set scRNA-seq data (L15–18), individual cell types in the cluster based on reference set scRNA-Seq data (original scRNA-seq dataset for L2, L3, L4, L5, L6, L7, L10, L11, L13, L14, SB1) were assigned and transferred to UMAP plot of test set scRNA-seq cluster using the label transfer method, which is the anchor-based label transfer implemented in the Seurat package. BM-MSCs (L15–18) were obtained from LONZA.

(B) Ratios of cells assigned to each reference set scRNA-seq cluster in each test set scRNA-seq cluster.

(C) Bar graphs showing the cell number of each BM-MSC line in cell subpopulation (test set scRNA-seq cluster), which were predicted as CL3 in (A) (left graph), and the VEGF secretion levels in four BM-MSC lines under ischemic condition (middle graph). Scatter plot shows Spearman's rank correlation coefficient between the rank of cell number of each BM-MSC line in CL3 (after anchor-based label transfer in test set scRNA-seq) and the rank of VEGF secretion level in each BM-MSC line under ischemic conditions (right graph). The *x*-axis represents the percentage of the number of cells in CL3 (after anchor-based label transfer in test set scRNA-seq) relative to the total number of cells captured by scRNA-seq in each cell line. The *y*-axis represents VEGF secretion in each cell line under ischemic conditions. Spearman's rank correlation (r_s = 0.63) indicated a positive correlation. In the case of n = 20 data points, the observed value of r_s must be more than 0.447 (positively correlated) to be considered significant (p-value < 0.05). r_s, Spearman's rank correlation coefficient.

(D) Expression of *LRRC75A* is represented using UMAP visualization (cell positions are from the UMAP plot of test set scRNA-seq). *LRRC75A* was more highly expressed in CL3 than in other clusters after anchor-based label transfer.

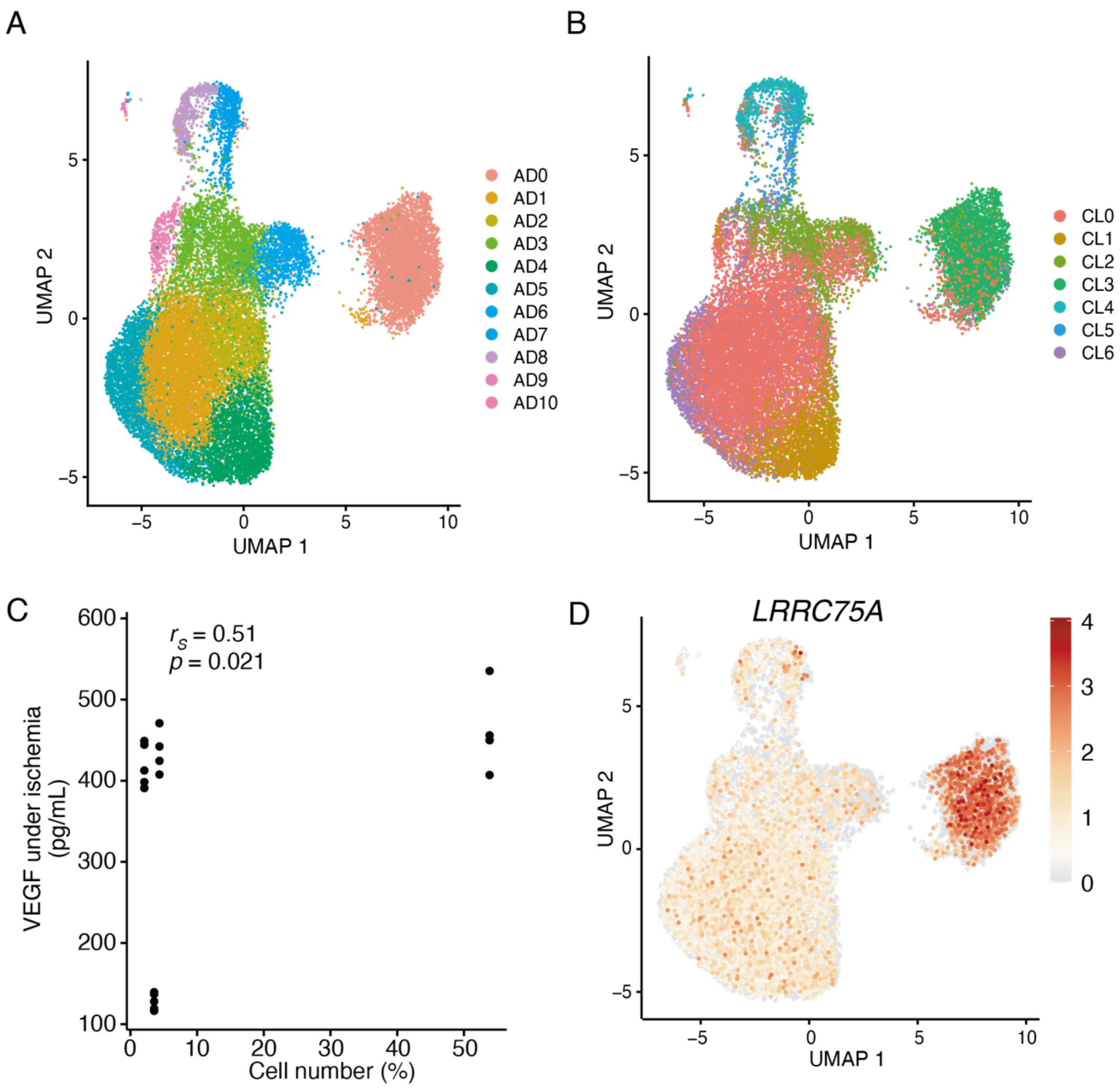

Supplementary Figure S6

Identification of CL3 in another scRNA-seq dataset (scRNA-seq dataset in adipose-derived multipotent mesenchymal stromal/stem cells (AD-MSCs))

(A) Visualization based on two-dimensional UMAP plotted as UMAP1 (*x*-axis) vs. UMAP2 (*y*-axis) for each cell type of four AD-MSC lines under normoxic conditions. Eleven clusters (AD0–11) marked with different colors were obtained using Seurat's graph-based clustering algorithm.

(B) Integrative analysis of reference set scRNA-seq data and AD-MSC scRNA-seq data. Similar to that shown in Supplementary Figure S5, to identify cell types in AD-MSC scRNA-seq data, individual cell types in the cluster based on reference set scRNA-seq data were assigned and transferred to UMAP plot of AD-MSC scRNA-seq cluster using label transfer method, which is the anchor-based label transfer implemented in the Seurat package.

(C) Scatter plot shows Spearman's rank correlation coefficient between the rank of cell number of each AD-MSC line in CL3 (after anchor-based label transfer in AD-MSC scRNA-Seq as shown in (B)) and the rank of VEGF secretion level in each AD-MSC line under ischemic conditions. The *x*-axis represents the percentage of the number of cells in CL3 (after anchor-based label transfer in test set scRNA-seq) relative to the total number of cells captured by scRNA-seq in each cell line. The *y*-axis represents VEGF secretion in each cell line under ischemic conditions. Spearman's rank correlation (r_s = 0.51) indicated a positive correlation. In the case of n = 20 data points, the observed value of r_s must be more than 0.447 (positively correlated) to be considered statistically significant (p-value < 0.05). r_s, Spearman's rank correlation coefficient.

(D) Expression of *LRRC75A* was represented by UMAP visualization (cell positions are from the UMAP plot of AD-MSC scRNA-seq). *LRRC75A* was more highly upregulated in CL3 than in other clusters after anchor-based label transfer.

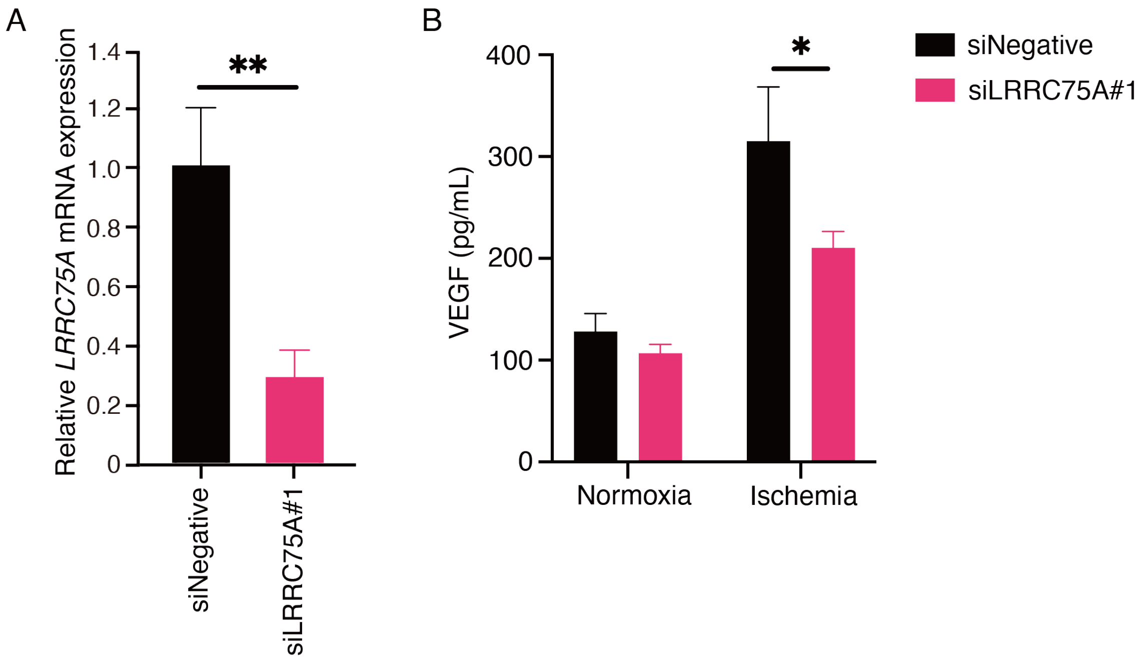

Supplementary Figure S7

Silencing of *LRRC75A* expression in adipose-derived multipotent mesenchymal stromal/stem cells (AD-MSCs) affects VEGF secretion induced by ischemic treatment. AD-MSCs were transfected with control siRNA (siNegative) (black bars) or *LRRC75A* siRNA (siLRRC75A#1) (magenta bars) under normoxic or ischemic conditions. *LRRC75A* mRNA (A) and VEGF secretion levels in the supernatant (B) were determined using qPCR and ELISA, respectively. All values indicate the mean ± standard error of mean of three independent biological replicates. (*$p < 0.05$, **$p < 0.01$, Student's *t*-test).

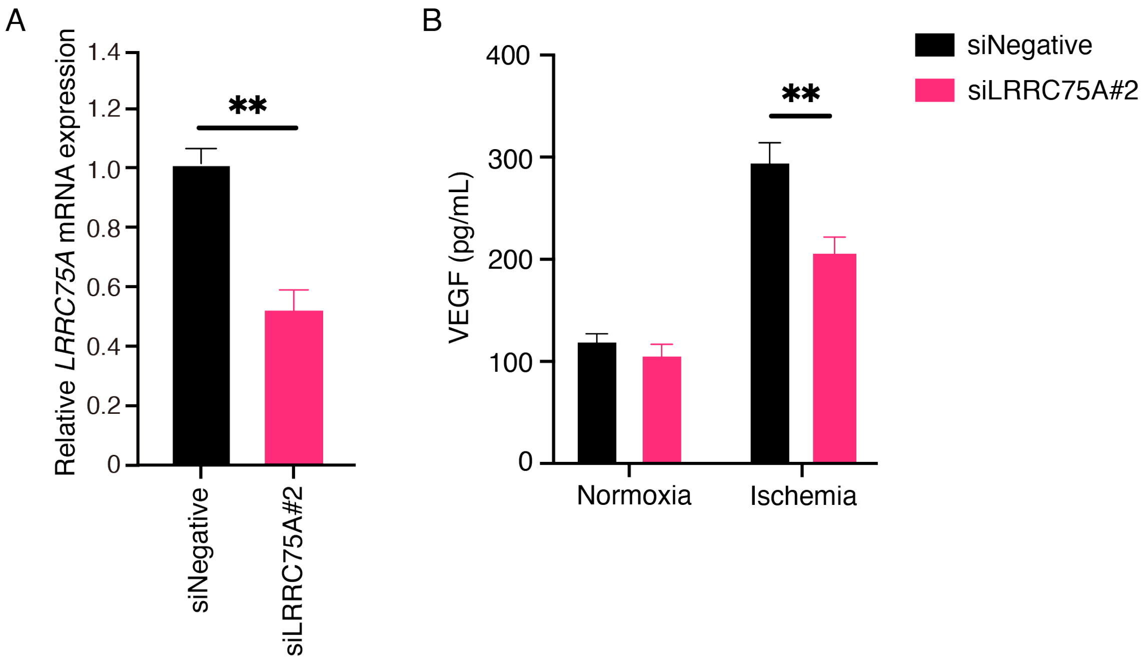

Supplementary Figure S8

Verification of siRNA results shown in Figure 4B, C using another siRNA.

As shown in Figure 4B, C, BM-MSCs were transfected with control siRNA (siNegative) (black bars) or *LRRC75A* siRNA (siLRRC75A#2) (magenta bars) under normoxic and ischemic conditions. *LRRC75A* mRNA (A) and VEGF secretion level in the supernatant (B) was determined using qPCR and ELISA, respectively. All values indicate the mean ± standard error of mean of three independent biological replicates. (**$p < 0.01$, Student's *t*-test).

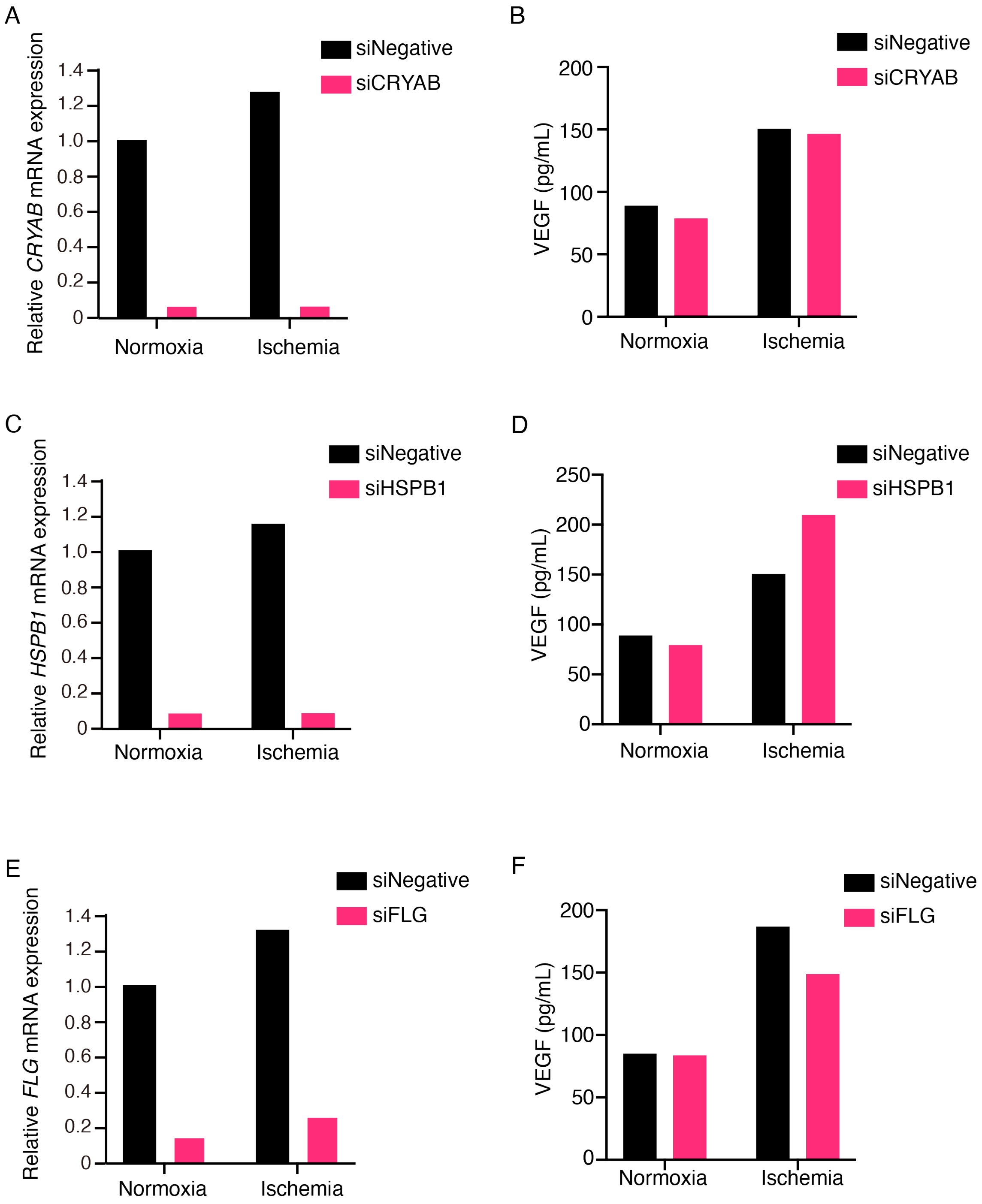

Supplementally Figure S9

Silencing *CRYAB*, *HSPB1*, or *FLG* expression did not affect the induction of VEGF secretion by ischemic treatment.

BM-MSCs were transfected with the indicated siRNAs: control siRNA (siNegative) (black bars), *CRYAB*, *HSPB1* and *FLG* siRNA (siCRYAB, siHSPB1, and siFLG) (magenta bars) under normoxic and ischemic conditions.

(A, C, E) To verify the efficiency of siRNA knockdown, the mRNA levels of *CRYAB* (A), *HSPB1* (C), and *FLG* (F) were determined using qPCR.

(B, D, F) VEGF secretion levels in BM-MSCs transfected with the indicated siRNAs were determined using ELISA.

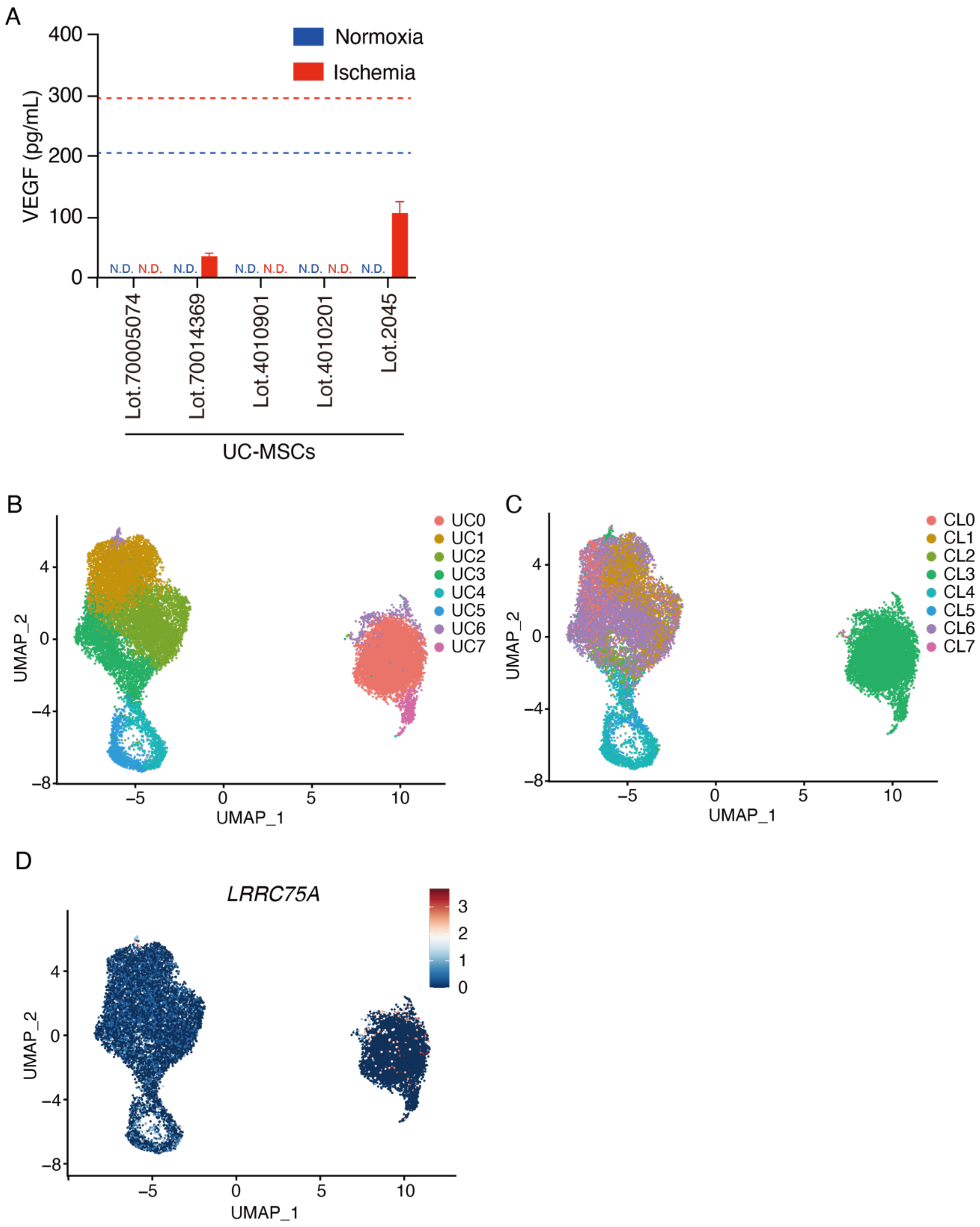

Supplementary Figure S10

Characteristics of umbilical cord (UC)-multipotent mesenchymal stromal/stem cells (MSCs).

(A) Variation in VEGF secretion among the five UC-MSC lines under normoxic and ischemic conditions. UC-MSCs were cultured under normoxic (blue) or ischemic (red) conditions for 16 hours. The level of VEGF secreted into the culture supernatant was determined using

ELISA. The amount of VEGF secretion is given as the mean ± standard error of the mean (*n* = 5 for each group). Dashed lines represent the lot-to-lot average value which calculated from the VEGF secretion levels of all BM-MSCs showed in Figure 1B (blue: normoxia, red: ischemia).

(B) Visualization based on two-dimensional uniform manifold approximation and projection (UMAP) plotted as UMAP1 (*x*-axis) vs. UMAP2 (*y*-axis) for each cell type of four UC-MSC lines under normoxic conditions. Eight clusters (UC0–7) marked with different colors were obtained using Seurat's graph-based clustering algorithm.

(C) Integrative analysis of reference set scRNA-seq data and UC-MSC scRNA-seq data. Similar to that shown in Supplementary Figure S5, to identify cell types in UC-MSC scRNA-seq data, individual cell types in the cluster based on reference set scRNA-seq data were assigned and transferred to an UMAP plot of UC-MSC scRNA-seq cluster using the label transfer method, which is the anchor-based label transfer implemented in the Seurat package.

(D) *LRRC75A* expression through UMAP visualization (cell positions are from the UMAP plot of UC-MSC scRNA-seq). Cells with high *LRRC75A* expression were remarkably few in CL3, classified by anchor-based label transfer.

Supplementary Table S1

Sequences of primers and probes used for real-time quantitative RT-PCR in this study.

Gene	Forward primer (5'-3')	Reverse primer (5'-3')	Probe (5'-3') *
LRRC75A	GGCAGCTGACATACCACCTCAG	CCCGACAGGTCCACTGTGTTG	CTGCCTCAAGGCTGTCCTGGCCGGAA
CRYAB	AGAGCGCCAGGATGAACATG	ACAGGGATGAAGTAATGGTGAGAG	CATCTCCAGGGAGTTCCACAGGAAATACCG
HSPB1	GCTGACGGTCAAGACCAAGG	GTAGCCATGCTCGTCCTGC	TCCTCGTGCTTGCCGGTGATCTCCA
FLG	AGAGCTGAAGGAACTTCTGGAAA	GGTCTATATCCAAGTGATCCATGAAG	CGGCAAATCCTGAAGAATCCAGATGACCCA

*Each probe was synthesized with a 6-FAM reporter dye and TAMRA quencher.
